# High-throughput phenotyping for non-destructive estimation of soybean fresh biomass using a machine learning model and temporal UAV data

**DOI:** 10.1186/s13007-023-01054-6

**Published:** 2023-08-26

**Authors:** Predrag Ranđelović, Vuk Đorđević, Jegor Miladinović, Slaven Prodanović, Marina Ćeran, Johann Vollmann

**Affiliations:** 1 Institute of Field and Vegetable Crops, Maksima Gorkog 30, 21000 Novi Sad, Serbia; 2 Faculty of Agriculture, Department of Genetics, Plant Breeding and Seed Science, University of Belgrade, Nemanjina 6, 11080 Zemun–Belgrade, Serbia; 3 Department of Crop Sciences, Institute of Plant Breeding, University of Natural Resources and Life Sciences, Konrad Lorenz Str. 24, 3430 Vienna, Tulln an der Donau, Austria

**Keywords:** High-throughput phenotyping, Biomass, Soybean, Machine learning, UAV

## Abstract

**Background:**

Biomass accumulation as a growth indicator can be significant in achieving high and stable soybean yields. More robust genotypes have a better potential for exploiting available resources such as water or sunlight. Biomass data implemented as a new trait in soybean breeding programs could be beneficial in the selection of varieties that are more competitive against weeds and have better radiation use efficiency. The standard techniques for biomass determination are invasive, inefficient, and restricted to one-time point per plot. Machine learning models (MLMs) based on the multispectral (MS) images were created so as to overcome these issues and provide a non-destructive, fast, and accurate tool for in-season estimation of soybean fresh biomass (FB). The MS photos were taken during two growing seasons of 10 soybean varieties, using six-sensor digital camera mounted on the unmanned aerial vehicle (UAV). For model calibration, canopy cover (CC), plant height (PH), and 31 vegetation index (VI) were extracted from the images and used as predictors in the random forest (RF) and partial least squares regression (PLSR) algorithm. To create a more efficient model, highly correlated VIs were excluded and only the triangular greenness index (TGI) and green chlorophyll index (GCI) remained.

**Results:**

More precise results with a lower mean absolute error (MAE) were obtained with RF (MAE = 0.17 kg/m^2^) compared to the PLSR (MAE = 0.20 kg/m^2^). High accuracy in the prediction of soybean FB was achieved using only four predictors (CC, PH and two VIs). The selected model was additionally tested in a two-year trial on an independent set of soybean genotypes in drought simulation environments. The results showed that soybean grown under drought conditions accumulated less biomass than the control, which was expected due to the limited resources.

**Conclusion:**

The research proved that soybean FB could be successfully predicted using UAV photos and MLM. The filtration of highly correlated variables reduced the final number of predictors, improving the efficiency of remote biomass estimation. The additional testing conducted in the independent environment proved that model is capable to distinguish different values of soybean FB as a consequence of drought. Assessed variability in FB indicates the robustness and effectiveness of the proposed model, as a novel tool for the non-destructive estimation of soybean FB.

**Supplementary Information:**

The online version contains supplementary material available at 10.1186/s13007-023-01054-6.

## Background

High-throughput phenotyping (HTP) allows that important information about cultivated plants be gathered in a faster and less expensive way than by standard techniques (manual measurements) used so far [1]. Great potential of HTP for in-season biomass estimation is not utilized in soybean, one of the most important oil crops in the world with annual global production > 350 million tons [2]. Biomass estimation offers comprehensive overview of a plant potential to use available recourses. This is crucial in low-input sustainable farming, where nutrients are mainly limited to natural resources and water supply depends on precipitation. Also, more robust genotypes intercept higher amount of light and have higher photosynthetic rates, which can increase seed yield. Low biomass accumulation in soybean leads to yield decrease due to reduced light interception and low radiation use efficiency [3]. In addition to the effect it has on yield, the amount and rate of accumulated biomass are also significant in weed management, which is one of the main tasks of a successful agricultural production, especially in organic farming systems [4]. Rapid canopy closure is also an important factor of weed suppression [5]. Therefore, biomass accumulation rate, as a measurement of growth, can contribute to a more efficient selection of superior genotypes during the breeding process. Having a tool for soybean biomass estimation at any time during the growing period would provide a new insight into crop development, thereby giving breeders continuous information on biomass accumulation. The determination of many important phenotypic traits within the current breeding programs is restricted due to complexity and lack of adequate tools [6]. A typical example of such a trait is plant biomass. Standard techniques for biomass assessment are destructive as well as time and labor-consuming. These difficulties can be overcome by using different remote sensing platforms and technologies.

Devices such as satellites or various UAVs use different sensors for collecting information about the Earth’s surface. Although satellites are becoming advanced and more precise, they cannot provide sufficient spatial resolution while data quality can be reduced due to clouds or other atmospheric factors [7]. On the other hand, the use of UAVs in agricultural research is growing year by year [8]. Photos taken by a digital camera mounted on UAV have a better resolution than those taken by satellites, thus ensuring greater data accuracy. The main advantage of using UAVs is in high-throughput i.e. a large amount of data collected in a short period of time [9, 10]. Hyperspectral (HS) imaging has the biggest potential for the assessment of different plant traits because HS cameras collect data from the entire spectrum [11]. The price of HS cameras is still too high compared to others such as RGB and multispectral (MS) sensors. These two are the most common camera types used in recording spectral reflectance of plant material in agriculture. Plant spectral reflectance is measured through values of digital numbers (DNs) which are used for the calculation of vegetation indices (VIs) linked to different plant traits [12–17]. Many VIs were used for biomass prediction in wheat [18], corn [19], and white oat [20]. In addition to the spectral reflectance sensed, data related to photogrammetry can also be obtained by processing UAV images through the structure from motion (SfM). Photogrammetry represents a technique that allows measuring the dimensions of the object on the image [21]. As a result of SfM processing of the UAV images a digital elevation model (DEM) can be obtained based on the point cloud data [22]. Other SfM derivatives such as the digital surface model (DSM) stand for surface elevation where the surface represents the plant canopy. In an agricultural cropping land, along DSM there is also a digital terrain model (DTM) which represents the ground-based elevation of bare soil. Based on the difference between DSM and DTM, it is possible to calculate plant height (PH) [23–25]. Another useful tool for PH determination is a light-detection and ranging system (LiDAR). This system measures the distance between the sensor and the object based on the time that passes when the laser signal goes from LiDAR to the object and back [26]. This method was used for PH assessment in sugarcane [27], wheat [28], or soybean and maize [29]. Even do LiDAR provides accurate measurements of plant traits in a non-destructive way there are some restrictions for wider application. Relatively high prices, complexity in data acquisition and data extraction are the main disadvantages of this system compared to the UAV camera sensors [30]. As an important indicator of plant growth, canopy cover (CC) can also be extracted as the percentage of plant pixels on an image [31]. Obtaining reliable information about PH and CC can be a useful tool in an estimation of accumulated biomass during the growing period.

Prediction of crop biomass can be based on a simple linear regression with different VIs [32], PH [33], or CC [34]. However, combining these individual data (predictors) can result in more accurate predictions. A study on barley shows that more precise results can be achieved by using both VIs and PH as opposed to using only reflectance-based data [35]. For plant trait estimation, the power of combining different predictors can be utilized through machine learning models (MLMs) and mathematical algorithms. One of the most popular algorithms based on classification and regression is random forest (RF) [36]. The RF was used for the prediction of corn [37] and soybean yields [38], leaf area index (LAI) of alfalfa, Rhodes grass, corn, carrots [39], and soybean [40], as well as for determination of wheat biomass [41]. For big data analysis, the most suitable methods are based on deep learning (DL) techniques [42]. These techniques use the artificial neural network (ANN), an ML algorithm designed to solve nonlinear relationships. The huge potential of DL models was utilized in many plant species for yield prediction [43], abiotic or biotic stress detection [44], leaf counting, and mutant classification [45]. Still, the RF was reported to be a better choice for biomass prediction compared to DL models, ANN and support vector regression (SVR), which was proven in wheat biomass estimation where RF outperformed ANN and SVR [46]. Partial least square regression (PLSR), another well-known MLM, is not as straightforward as RF. With PLSR, the dependent variable (crop trait) and independent variables (predictors) are explained through principal components. Different crop traits such as wheat yield [47] or yield and biomass of potato were successfully estimated using PLSR [48].

The aim of the study was to find an optimal model for the prediction of soybean fresh biomass (FB) using MLMs, VIs, CC and PH obtained by analyzing UAV digital images of different wavelength ranges. Moreover, the research objective was to estimate the robustness of the final model by conducting biomass screening of diverse soybean germplasm grown in different environments.

## Materials and methods

### Experimental site

The experiments were conducted in 2020 and 2021, at the experimental plots of the Institute of Field and Vegetable Crops in Rimski šančevi, Novi Sad, Serbia. For model calibration, ten soybean varieties with different maturity groups were sown in five replications in 2020 and four replications in 2021. The genotypes were sown on chernozem soil, characterized by homogeneous texture and well-aggregated structure. In total, 90 calibration plots, each 8 m^2^ were used for calibration of the biomass prediction model.

### UAV and data acquisition

The UAV used in the research was P4M (DJI, Shenzhen, China) equipped with six 1/2.9” CMOS sensors covering specific wavelength ranges. Five sensors were monochromatic B (450 ± 16 nm), G (560 ± 16 nm), R (650 ± 16 nm), Red edge (RE) (730 ± 16 nm), and Near-infrared (NIR) (840 ± 26 nm) and one RGB camera. Each sensor has a resolution of 2.08 megapixels (MP) with a focal length of 5.74 mm. During the flights, the UAV was connected with a real-time kinematic (RTK) system, a global navigation satellite system (GNSS) receiver, which provides centimeter-level precision of photographed objects on the image. The UAV is equipped with an integrated sun sensor which automatically corrects the reflectance based on the sunlight and secures data consistency in different weather conditions. Nine flights were conducted in this study, and the date for each of the flights was recorded in growing degree days (GDDs) calculated after emergence. In 2020, the photos were taken at 274, 413, 650, 745, and 1016 GDDs, and in 2021 at 215, 492, 747, and 1130 GDDs. Every flight was performed on a cloud-free, sunny day, wind speed didn’t exceed 10 m/s. The UAV shooting angle was course aligned, and image capture mode was set to equal time intervals while the front and side overlap of the images was 80%. Mission planning was done with DJI GS PRO software (DJI, Shenzhen, China). Flights were done at a 60 m altitude which secured the ground resolution of 3.17 cm/pixel. Subsequently, after each flight, soybean FB was harvested and measured with a specialized Wintersteiger combine. No significant amount of biomass was left on the field.

### Data processing

**Fig. 1:**
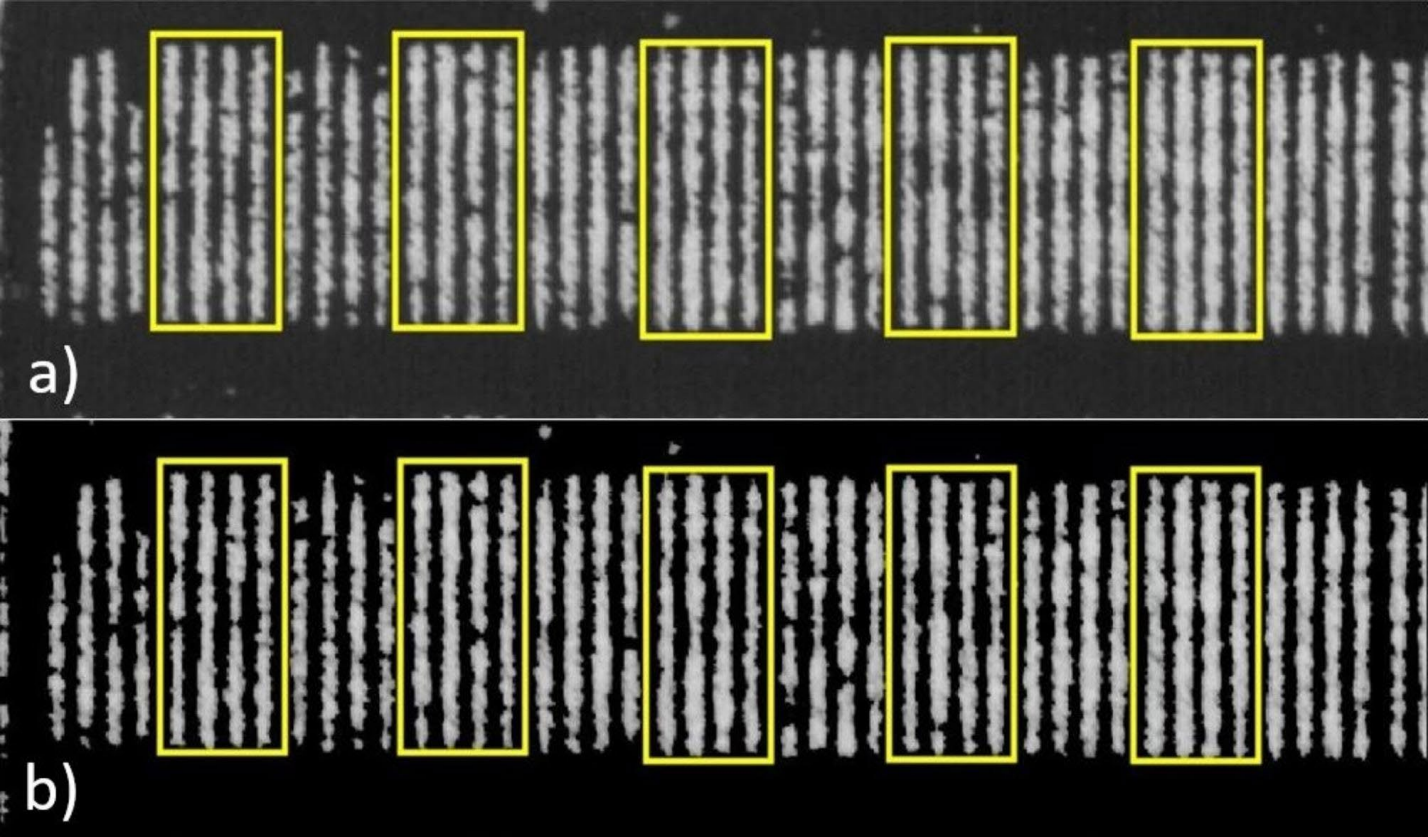
Region of interest (ROI) examples (yellow boxes) for individual soybean plots, (**a**) raw image of individual channel (as example NIR), (**b**) image after soil pixels were filtered out with the masking procedure

After collecting the photos of soybean genotypes, a dense cloud, digital elevation model (DEM) and orthomosaic were created using the Agisoft PhotoScan software (version 1.7.2. from 2021) build by Agisoft LLC from St. Petersburg, Russia (http://www.agisoft.com). The PH of the soybean genotypes was calculated using DEM (DSM and DTM), while CC and DNs were obtained from the orthomosaic for each plot. Each channel of the MS image was exported and analyzed using Fiji Is Just ImageJ (FIJI) software (version 1.51. from 2018), the open-source image analysis software [49]. First the region of interest (ROI) was created for every plot and then the masking procedure was performed to filter out the soil pixels (Fig. 1). The masking procedure was done in FIJI using the *Create Mask* function that eliminates soil and ensures that only plant pixels remain for further analysis. Following the masking procedure, the average DN value of each channel was exported together with the CC which was calculated as the percentage of plant pixels filling each ROI.

Based on the collected data, 31 different VI were calculated for each plot. The description and formula of each index are given in Additional file 1. High collinearity among many VIs was expected as they were obtained by combining the DNs of five spectral channels in different formulas. The relationship between VIs was analyzed so as to simplify the calculation within the MLM algorithm and ensure that collinearity does not disturb prediction quality. The correlation matrix was created in R with the *ggcorrmat* function from the *ggstatsplot* package [50]. This function creates the matrix plot based on the values of the correlation coefficient and marks non-significant relationships (p < 0.05). Highly correlated VIs were excluded using the *findCorrelation* function with ± 0.8 set as the cutoff value of pair-wise absolute correlation within the *Caret* package [51]. The function compares the mean absolute correlation (MAC) of two highly correlated VIs and eliminates the variable with the largest MAC.

**Fig. 2:**
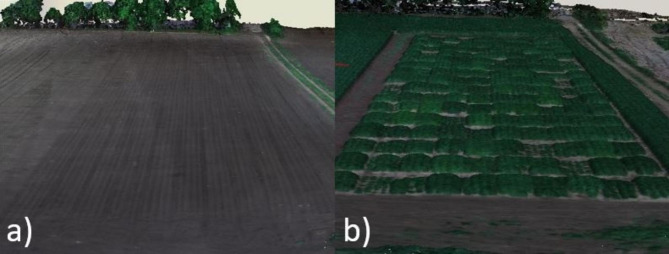
Example of (**a**) digital terrain model (DTM) and (**b**) digital surface model (DSM)

In both years, the PH of each plot was determined using the elevation models (DSM and DTM). The difference between DSM and DTM represents PH (Fig. 2). The average value of PH for each plot was used for further analysis.

### Machine learning models (MLMs)

The RF and PLSR were used to predict the soybean FB using CC, PH, and VIs. In the RF algorithm, the number of trees (*ntree*) was chosen by the lowest value of root mean square error (RMSE) while the number of predictors evaluated at each node (*mtry*) was selected based on the cross-validation. The RF was applied for the prediction of soybean FB using the *train* function from the *Caret* package with *mtry* = 3 and *ntree* = 500 set as optimal tuning parameters. A leave group out cross-validation (LGOCV) was implemented in the model where the harvested biomass and predictors from 70% of the randomly selected plots were used as a training set, while the remaining 30% were used as a test set. The LGOCV procedure was repeated 10 times, generating new training and test partitions in each cycle. The model performance was rated based on the average result of 10 predictions obtained through the LGOCV. For FB estimation with PLSR, the *Caret* package was also used including LGOCV approach. In the PLSR, an optimal number of latent variables was chosen based on the lowest value of RMSE in the estimation of a dependent variable (FB).

Prediction results of the models were evaluated through the coefficient of determination (R^2^), mean absolute error (MAE), and RMSE calculated with the following formulas:

1 $${R}^{2}={\left(\frac{\sum \left({x}_{i}-\overline{x}\right)\left({y}_{i}-\overline{y}\right)}{\sqrt{\sum {\left({x}_{i}-\overline{x}\right)}^{2}\sum {\left({y}_{i}-\overline{y}\right)}^{2}}}\right)}^{2}$$

2 $$MAE= \frac{\sum \left|{x}_{i}-{y}_{i}\right|}{N}$$

3 $$RMSE=\sqrt{\frac{\sum {\left({x}_{i}-{y}_{i}\right)}^{2}}{N}}$$

where *x*_*i*_ represents actual value of the trait for the *i-*th plot, $\bar x$-average of all actual values, *y*_*i−*_ predicted value of the trait for the *i-*th plot, $\bar y$-average of all predicted values and *N-* total plot number. The better performing MLM was chosen for further analysis.

### Evaluation of selected MLM on independent set of soybean genotypes

The proposed MLM for biomass prediction was additionally validated by performing temporal screening of soybean genotypes grown in different environments. The genotypes were divided into early (117) and late (89) based on the maturity group. They were sown on 8 m^2^ plots on sandy soil with low fertility and poor water retention to simulate a drought environment. As control groups, an identical set of genotypes was sown on a carbonate chernozem, a soil with favorable conditions, good water retention, and optimal soil fertility (Additional file 2). Trials for biomass screening were labeled as ED (early group grown under drought simulation), LD (late group grown under drought simulation), EC (early control), and LC (late control). For 206 soybean genotypes within ED, LD, EC, and ED trials, the necessary predictors were calculated from the UAV images collected in four-time points during 2020 and 2021. In both years, the trials were photographed at approximately 230, 390, 706, and 917 GDDs, with a difference of ± 1.8 – 21.6 GDDs. The FB of genotypes in ED, LD, EC, and LC trial was estimated at each time point.

## Results

### Development of the model for soybean FB estimation

As a result of plant growth, the values of CC, PH, and FB for soybean calibration plots increased as the season progressed in both years (Table 1).

**Table 1:** Average canopy cover – CC (%), plant height – PH (m), and fresh biomass – FB (kg/m^2^) measured on soybean calibration plots in 2020 and 2021.

*2020*				*2021*			
GDD (^o^C)	**CC (%)**	**PH (m)**	**FB (kg/m** ^**2**^ **)**	**GDD (** ^**o**^ **C)**	**CC (%)**	**PH (m)**	**FB (kg/m** ^**2**^ **)**
274	45.08	0.15	0.18	**215**	53.26	0.10	0.21
413	74.33	0.25	0.54	**492**	84.50	0.28	0.89
650	82.75	0.59	1.61	**-**	-	-	-
745	98.82	0.96	2.28	**747**	95.70	0.53	1.74
1016	87.87	0.99	3.03	**1130**	98.63	0.75	2.75

The results showed that soybean plants were taller in 2020 than in 2021, while almost a maximum of CC was achieved in both years. Still, in 2021 the CC remained high even at 1130 GDDs, while in 2020, it dropped over 10% between the last two measurements. The increase in biomass accumulation was also noticeable in both years.

**Fig. 3:**
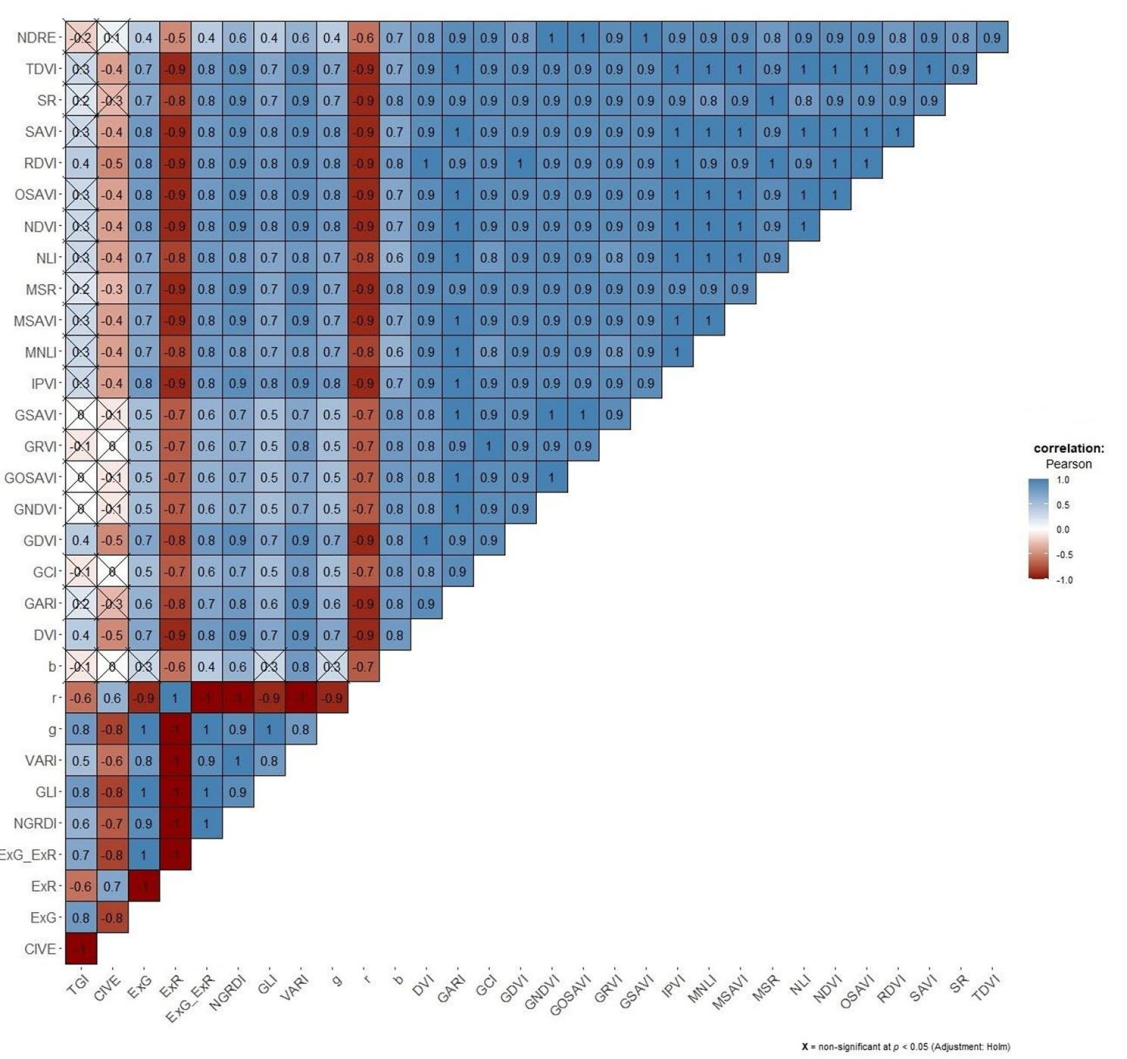
Correlation matrix for assessing the relationship between vegetation indices (VIs). Crosses on the plot indicate non-significant correlation coefficients (p < 0.05)

Correlation matrix revealed a strong relationship between many VIs, non-significant correlation coefficients (p < 0.05) were marked with cross (Fig. 3).

More than 62% (289/465) of all correlations were higher than the cut-off value set for pair-wise absolute correlation (± 0.8). Highly correlated variables were reduced by leaving only TGI and GCI as unique predictors. The relation between these two VIs was weak (r = – 0.1), while at the same time they showed the lowest MAC values when compared to the other VIs. For example, CIVE and GCI, or TGI and GNDVI, were not correlated (r = 0). Still, CIVE and GNDVI were excluded due to having a higher MAC than TGI and GCI.

### Biomass prediction models

**Fig. 4:**
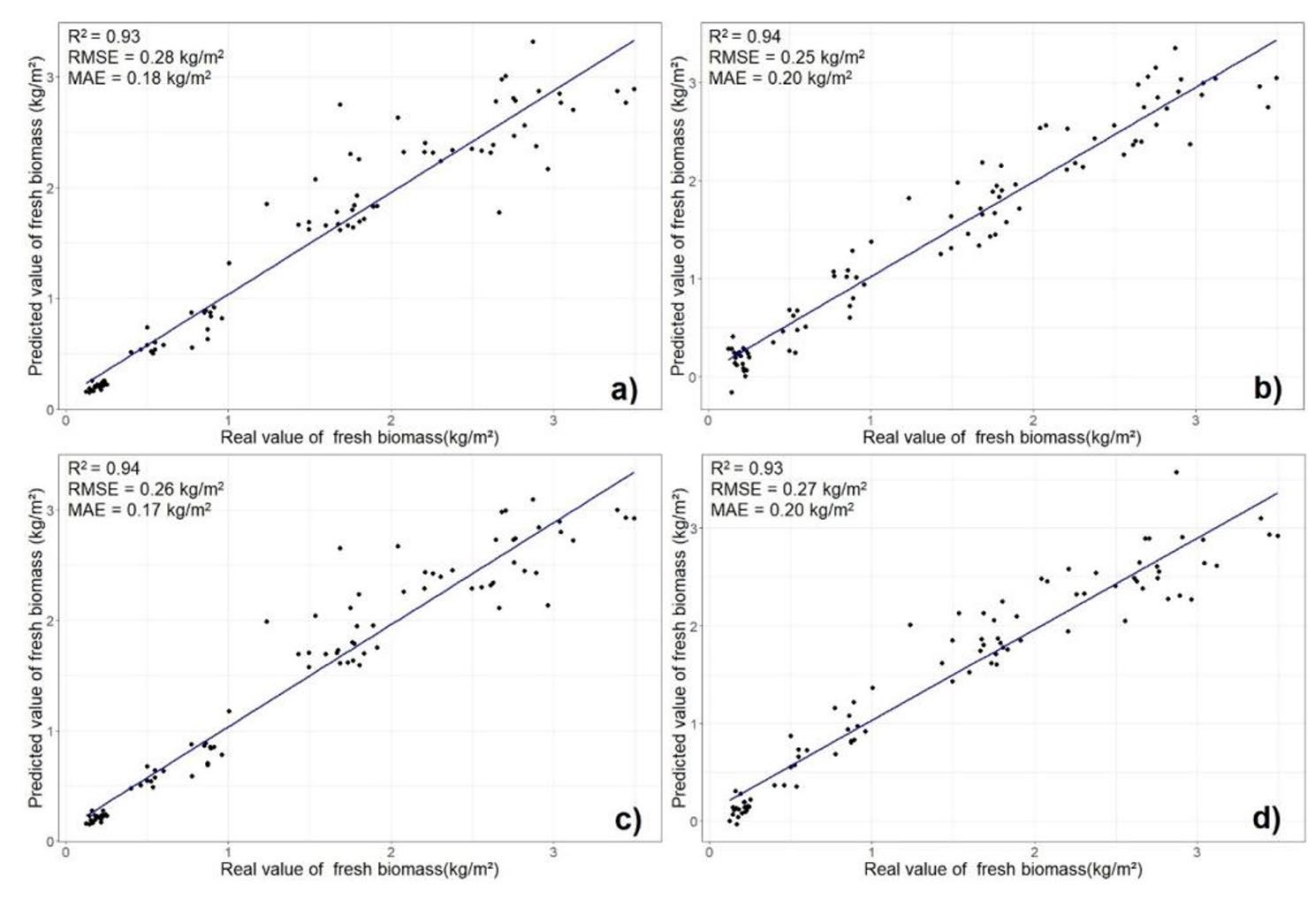
Correlation between the actual and the predicted fresh soybean biomass (kg/m^2^). (**a**) CC and PH combined with 31 VIs in RF, (**b**) CC and PH combined with 31 VIs in PLSR, (**c**) CC and PH combined with TGI and GCI in RF and (**d**) CC and PH combined with TGI and GCI in PLSR. Canopy cover – CC, plant height – PH, vegetation indices – VIs, random forest – RF, partial least squares regression – PLSR, coefficient of determination – R^2^, root mean square error – RMSE, and mean absolute error – MAE

Performance of the MLMs with different sets of predictors was analyzed by comparing the actual and the predicted values of soybean FB (Fig. 4).

There was a negligible difference in soybean FB when CC and PH were combined in models with all VIs, as opposed to being combined with TGI and GCI only. Further comparison of the MLMs was based on the results of the cross-validation for soybean FB prediction with a reduced set of predictors.

**Fig. 5:**
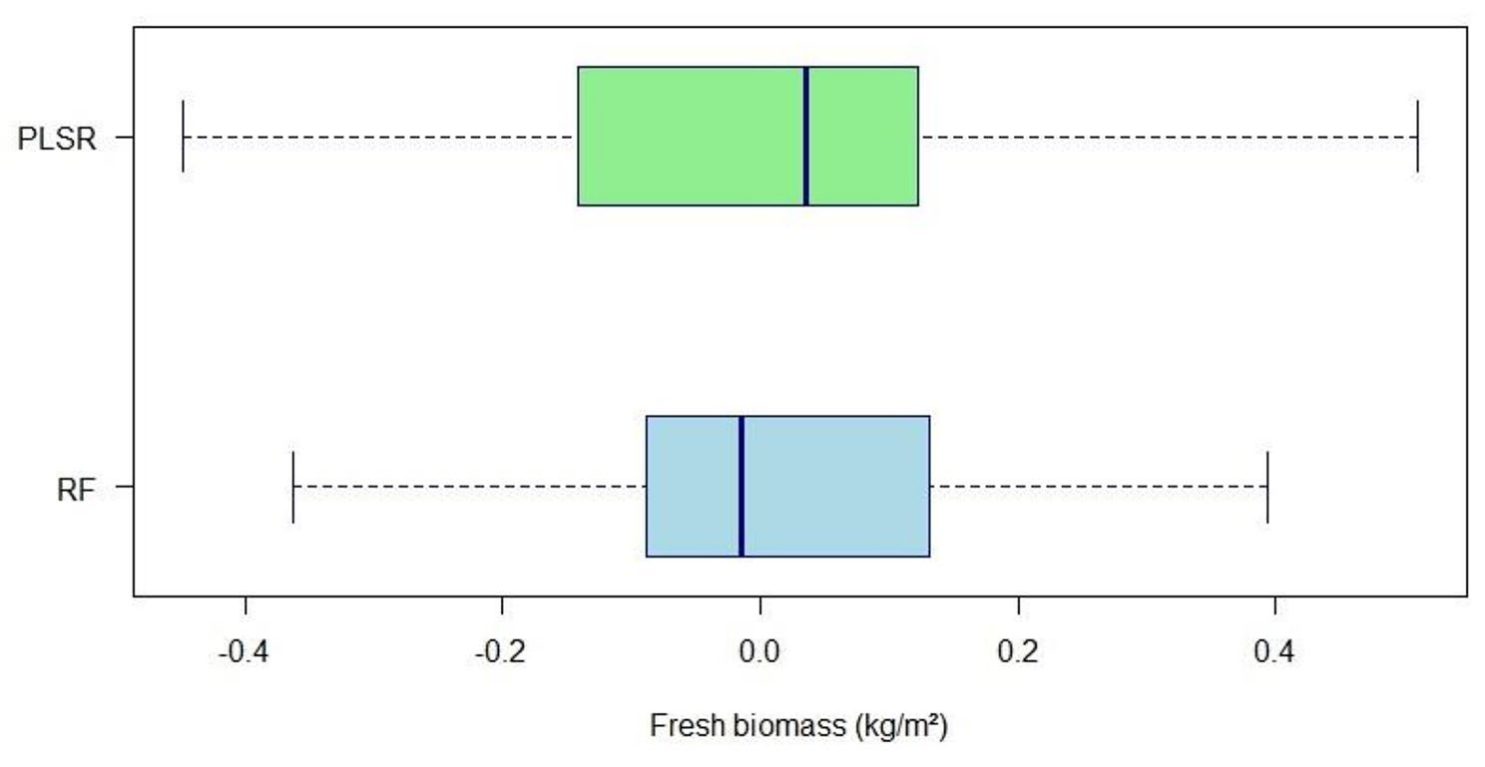
Box plots of differences between actual and predicted fresh soybean biomass (kg/m^2^) obtained with random forest (RF) and partial least squares regression (PLSR) with reduced set of predictors. The error bars show the 95% confidence interval while the line inside the boxes represent the median value

Both models showed good accuracy, as suggested by the high value of R^2^ and low RMSE and MAE. Nevertheless, RF provided slightly better results. The difference between the actual and predicted biomass was observed in the results of both models. Discrepancies were present in positive and negative directions. A lower standard deviation (SD) between the actual and the predicted values was obtained with the RF (SD = 0.25 kg/m^2^) model as compared to the PLSR (SD = 0.27 kg/m^2^) (Fig. 5).

**Fig. 6:**
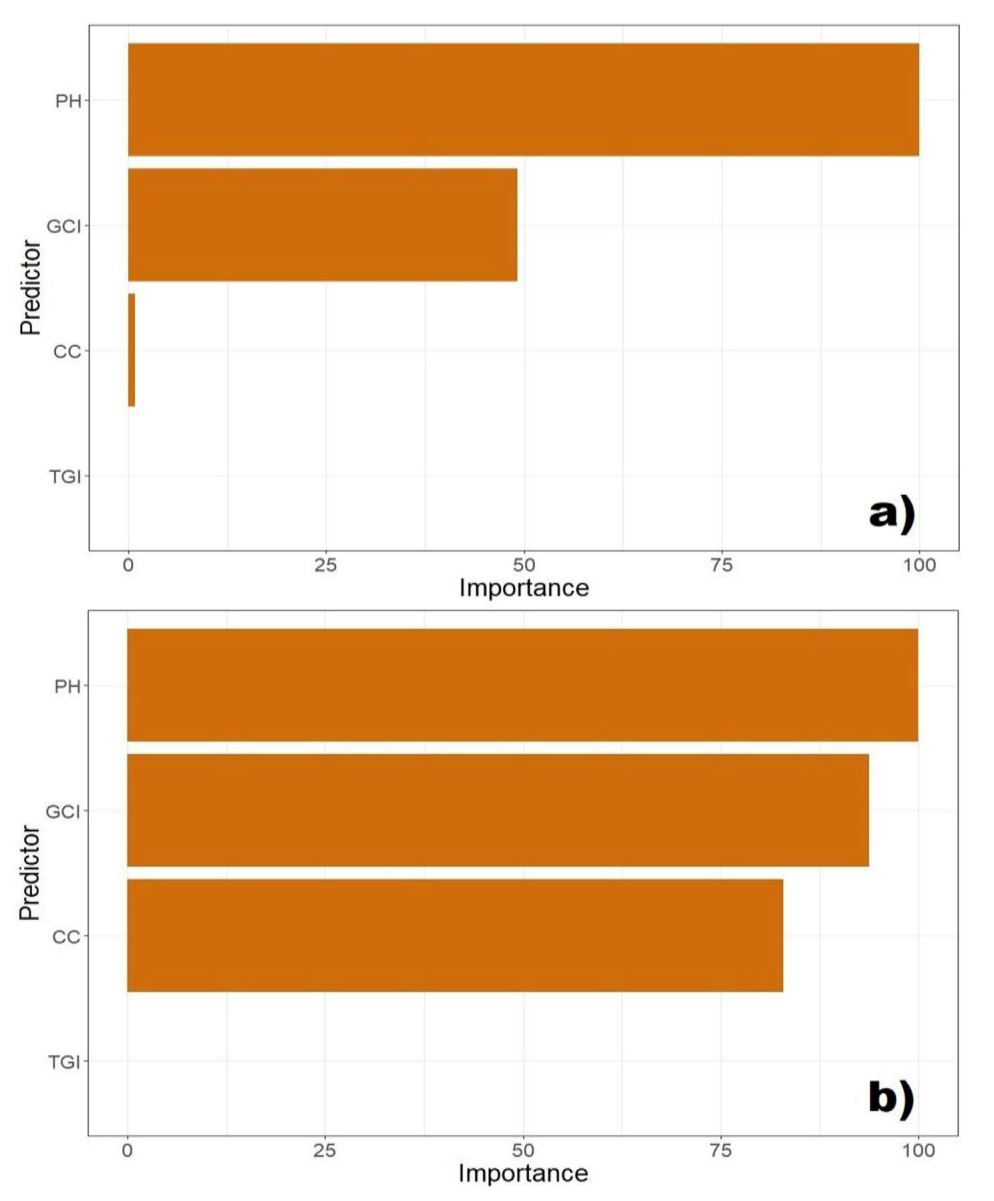
The importance of each predictor variable in (**a**) random forest (RF) and (**b**) partial least squares regression (PLSR) model for prediction of soybean fresh biomass (FB). Canopy cover – CC, plant height – PH, triangular greenness index – TGI, and green chlorophyll index – GCI

Even though the RF and PLSR have different mathematical algorithms, they used the same variables to predict the soybean FB. The importance of each predictor variable was extracted from the prediction models with the *varImp* function in the *caret* package and shown through the relative levels (0-100) (Fig. 6).

**Fig. 7:**
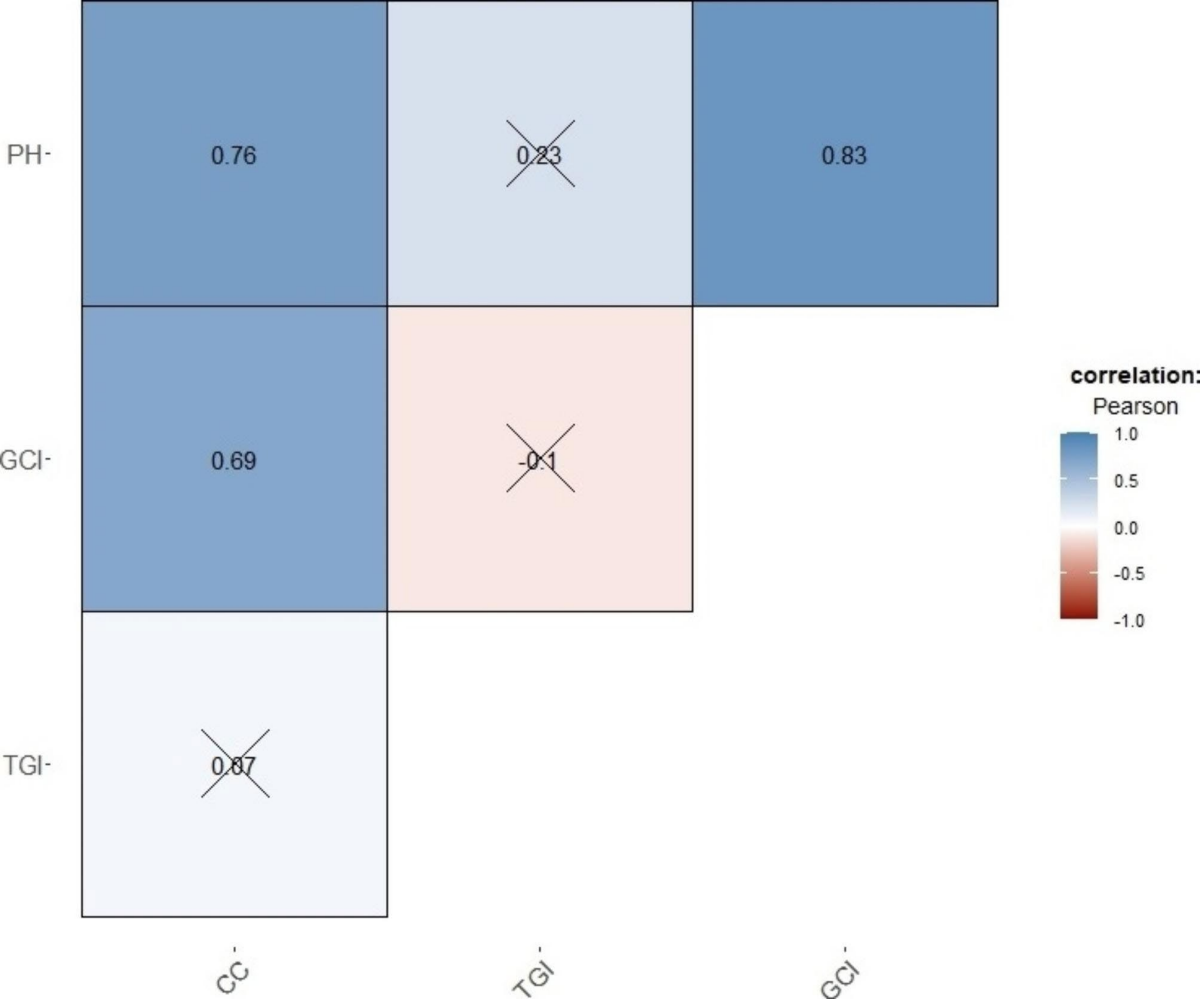
Correlation matrix for assessing the relationship between four selected predictor variables, plant height (PH), canopy cover (CC), green chlorophyll index (GCI), and triangular green index (TGI). Crosses on the plot indicate non-significant correlation coefficients (p < 0.05)

In both models, the predictor variables ranking was the same, the most important being PH, GCI, CC, and TGI, respectively. In the PLSR, the PH, GCI, and CC had a decisive impact in making the predictions, while the influence of TGI was marginal. The same situation with the TGI was found in the RF, where the CC also had a minor effect on the model performance. The GCI had a lower prediction effect in the RF as compared to the PLSR, while PH maintained its dominant position and was marked as a crucial variable. Correlation between four selected predictors was also calculated (Fig. 7).

Significantly high correlation was observed between PH, CC and GCI while no significance was observed between TGI and three other predictor variables.

### Temporal screening of soybean FB using proposed RF model

**Fig. 8:**
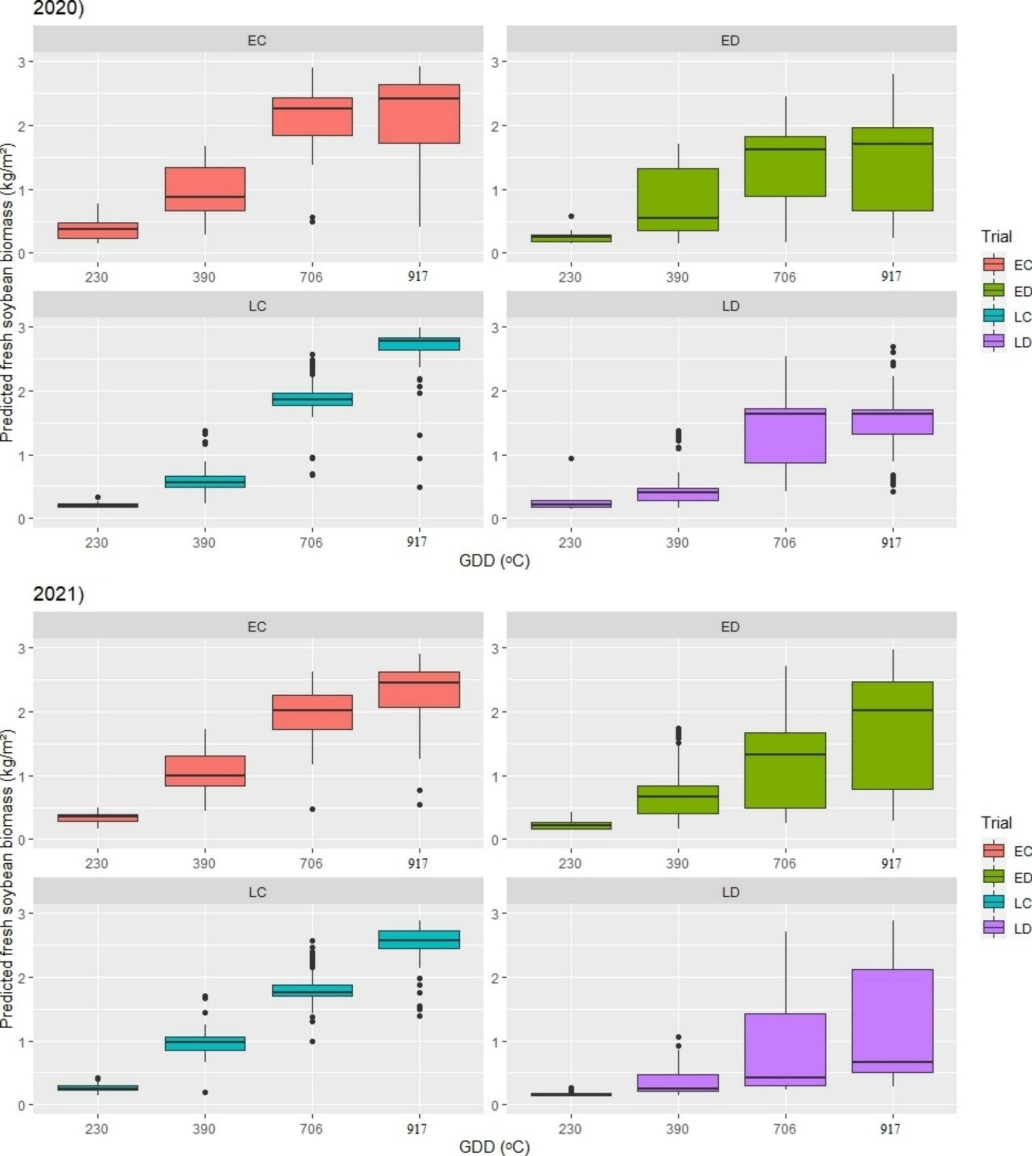
Temporal change in biomass accumulation for soybean genotypes grown in different environments based on the results of proposed random forest (RF) model. The line in each box plot stands for median value. The error bars represent the 95% confidence interval and outliers are represented by dots. Early group grown in drought simulation – ED, late group grown in drought simulation – LD, early control – EC and late control – LC, growing degree days after emergence – GDD (°C)

As a better-performing model, RF was subjected to further evaluation on an independent set of soybean genotypes grown in different environments. Biomass of 206 soybean genotypes was estimated within the ED, LD, EC, and LC trial in 2020 and 2021 (Fig. 8).

The results showed that the amount of accumulated organic matter increased throughout the season. Soybean FB was low at 230 GDDs, while the increase was noticeable after 390 GDDs. This pattern was present in all trials for both years. The unfavorable conditions did not affect soybean at the beginning of the growing period as much as it did later, when the negative effect of drought led to a decrease in biomass accumulation for genotypes in ED and LD as compared to the control.

## Discussion

The initial set of predictors contained PH, CC, and 31 VIs. Many VIs were highly correlated due to the similar origin, which enabled the reduction of their number without losing significant power in variability explanation. Further analysis suggested that only TGI and GCI could be used instead of a complete set of reflectance–based predictors. Although the number of VIs was reduced, it was necessary to check whether this reduction would affect the predictive ability of the RF and PLSR model. The results showed that the reduction of VIs did not have a significant effect on the performance of the MLMs. This indicates that soybean FB could be successfully predicted without using a large set of highly correlated VIs, which makes the entire process more efficient. A smaller error in prediction of FB was achieved with the RF model, thereby securing its advantage over PLSR as a novel tool for remote estimation of soybean biomass.

Even though the RF model had high accuracy, there were some differences between the predicted and the measured values of FB (RMSE = 0.26 kg/m²). The explanation for these discrepancies may lie in the predictors themselves. The PH stood out as the crucial variable which had the highest influence on the model’s performance. Some soybean genotypes are prone to lodging which can disturb the determination of plant PH in such a way that the predicted PH obtained by the analysis of DTM and DSM is lower than the actual one. As a result of the disturbance caused by lodging, imprecision in the remotely estimated biomass can be expected [52]. In the prediction of soybean FB, the effect of the two remaining VIs was not the same. The TGI had a low impact on the model’s performance, while in the case of GCI the results were a little different. The significance of GCI lies in its essence, as it was created by a combination of G and the particularly important NIR channel, associated with biomass in the previous studies [53]. On the other hand, the TGI is based on plant reflectance caused by light from the visible part of the spectrum. This part of light spectrum does not penetrate plant tissue as deeply as NIR [54, 55] does, which could be the reason why the GCI was far more important for biomass prediction than TGI. This is especially significant in later development stages, when plants achieve high CC and PH with lots of interlaced leaves. Adding more NIR–based VIs would not improve model accuracy because all VIs were highly correlated (r > 0.8) with the used GCI. Finally, the model’s precision can be disturbed by weeds if their presence leads to an increase in CC. Also, weeds leaf tissue can cause changes in spectral reflectance of the plots which can harm precise determination of selected VIs. In that case, the model could overestimate biomass for that plot. This can be expected with PLSR, where CC and GCI has a great influence. In the proposed RF model, the importance of GCI and especially CC is lower, ensuring more stable predictions of soybean FB regardless of canopy density.

In the barley research, biomass prediction relied on the correlation with PH, resulting in R^2^ = 0.72 [56]. Plant PH was also obtained using SfM and DSM in calculations of the crop surface model (CSM), thus ensuring higher accuracy compared to ground measurements. Biomass estimation based solely on PH simplifies the process, but it can also be very challenging. In the barley study, no other variables could compensate for the shortcomings of one predictor prone to a lodging error such as PH. On the other hand, utilization of the VIs, PH, and CC as combined set of predictors requires more computing, but it provides better results in biomass prediction. For instance, tomato fresh shoot mass was predicted several times during the growing season using the RF algorithm and a set of combined predictors [57]. Six VIs, including G – R index [58], NDRE and different variations of NDVI, were used alongside other predictors such as plant area, length, width or PH. This approach secured high accuracy in the estimation of tomato biomass with R^2^ = 0.88. The VIs (especially the G–R index) were very important in making the prediction, but the crucial variable was plant area. Contrary to the study on soybean where PH was the main predictor, for tomato plant PH was not significant. This could be related to the different growth type of soybean and tomatoes. Unlike soybean, which has a predominantly vertical growth, tomatoes mainly spread their shoots horizontally, causing a smaller PH variation between the plants. The PH was also highly ranked in the prediction of maize biomass using different MLMs [59]. In the research on maize, the initial set of predictors containing different VIs, PH and volumetric parameters was reduced because some variables were highly correlated. This was done to eliminate the possible impact of multicollinearity on the model’s predictive ability, in the same way as it was done in the prediction of soybean FB. All of the above suggests that the selection of a proper set of predictor variables customized to the certain plant shoot architecture is crucial for successful biomass estimation. This was given special attention in the proposed RF algorithm for the determination of soybean FB, which therefore resulted in high accuracy (R^2^ = 0.94).

The quality of the proposed model was additionally tested in a two–year trial where FB was predicted for 206 soybean genotypes. The results obtained on the evaluation plots in 2020 and 2021 showed that both early and late genotypes from ED and LD trials accumulated less FB than the control. The reduction in biomass as the consequence of unfavorable conditions was expected based on the previous studies on soybean and corn [60, 61]. The negative impact of drought on soybean development manifested itself through reduced PH and LAI [62]. Moreover, the water deficit changed spectral reflectance of the soybean plants, causing an increase in visible light reflection, while at the same time NIR dropped [63]. This means that values of TGI and GCI were also modified, as they directly depend on canopy reflectance. The proposed model for remote estimation of soybean FB recognized changes in predictor variables, which was proven by the variability of predicted results in the testing environments. The difference in the estimated FB was especially noticeable after 390 GDDs for both the early and late genotypes, because soybean is less sensitive to drought in the early development stages while the greatest damage occurs after flowering, i.e. the generative phase [64]. Furthermore, according to the results, the late genotypes accumulated more biomass than the early ones, which was anticipated as a result of the longer growing period. All of the above confirms the robustness of the proposed RF model, based on its ability to distinguish different values of soybean FB not just between different environments (EC/ED and LC/LD), but also within each environment.

## Conclusions

The estimation of soybean FB was tested using reflectance and photogrammetry based predictors in two different MLMs, including RF and PLSR. More precise results were obtained using the RF model with only four predictors. The PH and GCI stood out as the most important variables respectively, while the impact of CC and TGI was minor. The proposed MLM showed that the soybean FB can be accurately estimated (R^2^ = 0.94) using a small set of predictors. The reduction in the number of VIs from the initial 31 to just two did not affect model performance. This information can be very useful for the future studies aiming to reduce unnecessary calculations. The robustness of the MLM was demonstrated on divergent soybean germplasm in drought simulation environments, where the predictor variables were affected by the unfavorable growing conditions. Based on these changes, the model adjusted the results of soybean FB prediction between as well as within environments. The results of additional testing proved that the model is able to adapt to different conditions which is important for gathering significant information about biomass accumulation and soybean development. This information could be utilized by practice and science. The farmers may benefit from it by knowing the current status of the crop biomass production and managing the production processes based on the obtained results. On the other hand, scientists from different fields could find this prediction model interesting as a tool for the enhancement of their research. For example, the proposed HTP model can provide a huge amount of data that can be used as new traits in soybean breeding programs. This can result in a more efficient and more precise selection of the best varieties. Still, there is a possibility for additional adjustments of the model. The observed significant correlation between PH, GCI, and CC indicates that further improvement of the proposed model could be achieved through enhanced predictor selection. To realize this idea, the additional testing of the model (new environment and germplasm) and literature survey will be continued in the future to collect as much information as possible. The acquired data will be used to perceive the possibilities for enhancement of the proposed model for soybean FB prediction.

### Electronic supplementary material

Below is the link to the electronic supplementary material.

**Additional file 1**. Vegetation indices (VI’s). R, G, B, RE, and NIR-digital numbers of each channel from digital UAV photo.

**Additional file 2**. Mechanical composition, % of organic matter, and water retention capacity of the soil where experiments were set up to additionally test the proposed model for soybean biomass prediction. ED (early group grown in drought simulation), LD (late group grown in drought simulation), EC (early control), and LC (late control).

## Data Availability

The raw data used in this study are available from the corresponding author on reasonable request.
