# Supplementary material for: High-throughput phenotyping for non-destructive estimation of soybean fresh biomass using a machine learning model and temporal UAV data
Source: Plant Methods. 2023 Aug 26;19:89. doi: 10.1186/s13007-023-01054-6 (PMC10463513; doi:10.1186/s13007-023-01054-6)
Supplement: Supplementary file 1 — Additional file 1. Vegetation indices (VI’s). R, G, B, RE, and NIR-digital numbers of each channel from digital UAV photo. [file 13007_2023_1054_MOESM1_ESM.docx]

Additional file 1. Vegetation indices (VI’s). R, G, B, RE and NIR-digital numbers of each channel from digital UAV photo.

| Vegetation index | Name | Formula |
| --- | --- | --- |
| r | Normalized red | $\frac{R}{R+G+B}$ |
| g | Normalized green | $\frac{G}{R+G+B}$ |
| b | Normalized blue | $\frac{B}{R+G+B}$ |
| GLI | Green leaf index | $\frac{2*G-R-B}{2*G+R+B}$ |
| VARI | Visible atmospherically resistant index | $\frac{G-R}{G+R-B}$ |
| NGRDI | Normalized green red difference index | $\frac{G-R}{G+R}$ |
| TGI | Triangular greenness index | $G-0.39*R-0.61*B$ |
| CIVE | Color index of vegetation extraction | $0.441*R-0.811*G-0.385*B-18.78745$ |
| ExG | Excessive green | $2*g-r-b$ |
| ExR | Excessive red | $1.4*r-g$ |
| ExG-ExR | Excess green minus excess red index | $ExG-ExR$ |
| DVI | Difference vegetation index | $\frac{NIR}{R}$ |
| GARI | Green atmospherically resistant vegetation index | $\frac{NIR-(G-1.7*\left( B-R \right))}{NIR+(G+1.7*\left( B-R \right))}$ |
| GCI | Green chlorophyll index | $\frac{NIR}{G}-1$ |
| GDVI | Green difference vegetation index | $NIR-G$ |
| GNDVI | Green normalized difference vegetation index | $\frac{NIR-G}{NIR+G}$ |
| GOSAVI | Green optimized soil adjusted vegetation index | $\frac{NIR-G}{NIR+G+0.16}$ |
| GRVI | Green ratio vegetation index | $\frac{NIR}{G}$ |
| GSAVI | Green soil adjusted vegetation index | $1.5*\frac{NIR-G}{NIR+G+0.5}$ |
| IPVI | Infrared percentage vegetation index | $\frac{NIR}{NIR+R}$ |
| MNLI | Modified non-linear vegetation index | $\frac{1.5*(NIR^{2}-R)}{0.5*(NIR^{2}+R)}$ |
| MSAVI | Modified soil adjusted vegetation index | $\frac{2*NIR+1-\sqrt{\left( 2*NIR+1 \right)^{2}-8*(NIR-RED)}}{2}$ |
| MSR | Modified simple ratio | $\frac{\frac{NIR}{R}-1}{\sqrt{\frac{NIR}{R}+1}}$ |
| NLI | Non-linear vegetation index | $\frac{NIR^{2}-R}{NIR^{2}+R}$ |
| NDVI | Normalized difference vegetation index | $\frac{NIR-R}{NIR+R}$ |
| OSAVI | Optimized Soil Adjusted vegetation index | $\frac{NIR-R}{NIR+R+0.16}$ |
| RDVI | Renormalized Difference vegetation index | $\frac{NIR-R}{\sqrt{NIR+R}}$ |
| SAVI | Soil adjusted vegetation index | $\frac{1.5*(NIR-G)}{0.5*(NIR+G)}$ |
| SR | Simple ratio index | $\frac{NIR}{R}$ |
| TDVI | Transformed difference vegetation index | $\frac{1.5*(NIR-R)}{\sqrt{NIR^{2}+R+0.5}}$ |
| NDRE | Normalized difference red-edge | $\frac{NIR-RE}{NIR+RE}$ |
