# Supplementary material for: High-throughput phenotyping for non-destructive estimation of soybean fresh biomass using a machine learning model and temporal UAV data
Source: Plant Methods. 2023 Aug 26;19:89. doi: 10.1186/s13007-023-01054-6 (PMC10463513; doi:10.1186/s13007-023-01054-6)
Supplement: Supplementary file 2 — Additional file 2. Mechanical composition, % of organic matter, and water retention capacity of the soil where experiments were set up to additionally test the proposed model for soybean biomass prediction. ED (early group grown in drought simulation), LD (late group grown in drought simulation), EC (early control), and LC (late control). [file 13007_2023_1054_MOESM2_ESM.docx]

Additional file 2. Mechanical composition, % of organic matter and water retention capacity of the soil where experiments were set up to additionaly test the proposed model for soybean biomass prediction. ED (early group grown in drought simulation), LD (late group grown in drought simulation), EC (early control), and LC (late control).

| Year | Trial | Coarse sand %  2-0.2 mm | Fine sand %  0.2-0.02 mm | Silt %  0.02-0.002 mm | Clay %  <0.002 mm | Organic matter % | Water retention (% vol.) | | |
| --- | --- | --- | --- | --- | --- | --- | --- | --- | --- |
|  |  |  |  |  |  |  | 0.33 bar | 6.25 bar | 15 bar |
| 2020 | ED | 1.31 | 72.53 | 14.28 | 11.88 | 0.99 | 14.16 | 7.49 | 6.41 |
|  | LD | 0.77 | 64.87 | 19.08 | 15.28 | 1.34 | 16.90 | 8.92 | 7.73 |
|  | EC | 0.88 | 46.80 | 28.16 | 24.16 | 2.57 | 26.32 | 15.7 | 14.54 |
|  | LC | 0.96 | 42.24 | 30.84 | 25.96 | 2.73 | 27.15 | 16.04 | 14.54 |
| 2021 | ED | 0.70 | 60.82 | 19.4 | 19.08 | 1.93 | 21.58 | 11.10 | 9.56 |
|  | LD | 0.78 | 68.10 | 15.96 | 15.16 | 1.33 | 17.97 | 9.20 | 7.74 |
|  | EC | 0.46 | 44.26 | 24.28 | 31.00 | 2.67 | 28.71 | 17.82 | 15.88 |
|  | LC | 0.41 | 44.91 | 27.04 | 27.64 | 2.45 | 28.04 | 17.10 | 15.04 |
